# Acknowledgement to Reviewers of *Biology* in 2015

**DOI:** 10.3390/biology5010008

**Published:** 2016-01-22

**Authors:** Biology Editorial Office

**Affiliations:** MDPI AG, Klybeckstrasse 64, CH-4057 Basel, Switzerland; biology@mdpi.com

The editors of *Biology* would like to express their sincere gratitude to the following reviewers for assessing manuscripts in 2015. 

We greatly appreciate the contribution of expert reviewers, which is crucial to the journal’s editorial decision-making process. Several steps have been taken in 2015 to thank and acknowledge reviewers. Good, timely reviews are rewarded with a discount off their next MDPI publication. By creating an account on the submission system, reviewers can access details of their past reviews, see the comments of other reviewers, and download a letter of acknowledgement for their records. This is all done, of course, within the constraints of reviewer confidentiality. Feedback from reviewers shows that most see their task as a voluntary and mostly unseen work in service to the scientific community. We are grateful to our reviewers for the contribution they make.

Abelli, L.
Abernethy, Katharine
Andersson, Emma
Andreetta, Anna
Archambault, Steven
Bajoghli, Baubak
Barreda, Daniel
Beaujean, Nathalie
Bejarano, Eduardo R.
Benfield, David A.
Bernhard, Frank
Blackstone, Neil
Bonvin, Alexandre M.J.J.
Boto, Luis
Botvinick, Elliot L.
Breinbauer, Rolf
Buchmann, Kurt
Canapa, Adriana
Chak, Kayam
Cohen, Mark L.
Coll, Julio
Craw, Pascal
Dacks, Joel B.
Dalmo, Roy A
Dave, Kunjan R.
De Bernonville, Thomas Dugé
De Jong, Jill L.O.
Demyanets, Svitlana
Diamond, Gill
Doherty, James M.
Dooley, Helen
Dunn, Ryan J. K.
Elks, Philip
Eppler, Elisabeth
Esteban, Maria Angeles
Ferbeyre, Gerardo
Fernandez, Pedro Alexandrio
Fonken, Laura K.
Foote, Lee
Forlenza, Maria
Fox, Jennifer L.
Froeyen, Matheus
Fukunishi, Yoshifumi
Gadella, Bart M.
Gallicano, G. Ian
Garcia De La Torre, Jose
García-Ayala, Alfonsa
Giordano, Mario
Glazier, Douglas S.
Glynn, Paul
Goldring, Mary
Gomes Rocha, Agostinho
Gomez, David
Granger, David
Gutiérrez Martín, Santiago
Hasselquist, Eliza Maher
Henry, Morgane
Herms, Dan
Hilchie, Ashley
Hirase, Hajime
Hiroaki, Hidekazu
Hoeck, Joerg D.
Holmes, Dawn E.
Howl, John
Ishii, Tomohiro
Ivarsson, Ylva
Jaudszus, Anke
Jorgensen, Jorunn
Kaiyala, Karl J.
Kamiya, Masakatsu
Kang, Cuijie
Kaster, Jerry
Kianoush, Nima
Kim, Jae Ho
Kima, Peter E.
Kishigami, Satoshi
Knudsen, Kerry
Kohda, Takashi
Kolmar, Harald
Korytowski, Witek
Kuwahara, Masayasu
Langeveld, Jan
Lannig, Gisela
Larson-Prior, Linda J.
Lee, Hong Kyu
Legname, Giuseppe
Lewy, Alfred J.
Liu, Guosheng
Loizides, Ursula
Luchinat, Claudio
Lücking, Robert
Maccani, Matthew
Martin, Sam
Martin, William
Masters, Colin L.
McCune, Amy
Mckenzie, Deb
Midzak, Andrew
Miyata, Naoyuki
Miyata, Seiji
Moore, Sean D.
Mulero, Victor
Nickels, Joseph T
Nicoletti, Claudio
Nomiyama, Hisayuki
Ohta, Yuko
Owczarzy, Richard
Pandya, Chetanya
Papaleo, Elena
Paradis, Emmanuel
Pavlicev, Mihaela
Pavlova, Elena
Peirson, Stuart N
Pfaller, M. A.
Piercey-Normore, Michele
Pines, Ophry
Pirro, Valentina
Potempa, Lawrence A.
prasanth Koppolu, Bhanu
Putzke, Aaron
Rahimi, Nader
Rakers, Sebastian
Reading, Benjamin J.
Rensen, Patrick C.N.
Rice, Charles D.
Rijkers, Ger
Rodino-Klapac, Louise R.
Rouault, Tracey A.
Rozemuller, A.j.m.
Ruehl, Ralph
Russell, Mark W.
Ryan, Mike
Salinas, Irene
Santacroce, Luigi
Schebb, Nils Helge
Shi, Rui
Shindou, Hideo
Sills, Deborah L.
Stafford, James L.
Sutton-McDowall, Melanie L.
Tamura, Koji
Tan, Dun-Xian
Tani, Shuji
Tehlivets, Oksana
Thompson, Sam
Tomlinson, Sean
Tranulis, Michael A.
Uchiyama, Susumu
Uzawa, Takanori
Van Kemenade, B.M. Lidy
Vickers, Kasey C.
Vinknes, Kathrine J.
Wadley, Alex
Wang, Zejing
Wenderoth, Nicole
White, Craig
Wiechec, Emilia
Wiegertjes, Geert
Williams, Thomas L.
Wilson, Melinda E.
Winge, Dennis R.
Xu, Junjie
Yeh, Shyi-Dong
Yu, Juehua
Zacharias, Martin
Zeitzer, Jamie M.
Zou, Jun
Zwollo, Patty

